# The Impact of Culturally-Centered Care on Peripartum Experiences of Autonomy and Respect in Community Birth Centers: A Comparative Study

**DOI:** 10.1007/s10995-021-03245-w

**Published:** 2021-11-24

**Authors:** Jennifer I. Almanza, J.’Mag Karbeah, Katelyn M. Tessier, Carrie Neerland, Kathrin Stoll, Rachel R. Hardeman, Saraswathi Vedam

**Affiliations:** 1 Department of OBGyn, University of Minnesota Medical School, 606, 24th Avenue South, Suite 300, Minneapolis,MN 55454, USA; 2 1002 Livingston Ave, West St. Paul, MN 55118, USA; 3 Population Health Sciences Predoctoral Trainee, Division of Health Policy Management, University of Minnesota School of Public Health, 420 Delaware St SE MMC 729, Minneapolis, MN 55455, Canada; 4 Masonic Cancer Center, Biostatistics Core, University of Minnesota, 717 Delaware St SE, Minneapolis, MN 55455,USA; 5 University of Minnesota School of Nursing, 5-140 Weaver Densford Hall, 308 Harvard St SE, Minneapolis, MN 55455,USA; 6 Birth Place Lab, Department of Family Practice, Faculty of Medicine, University of British Columbia, 304-5950 University Boulevard, Vancouver, BC V6K 1N3, Canada

**Keywords:** BIPOC, Birth center, Equity, Respect, Autonomy

## Abstract

**Objective:**

National studies report that birth center care is associated with reduced racial and ethnic disparities and reduced experiences of mistreatment. In the US, there are very few BIPOC-owned birth centers. This study examines the impact of culturally-centered care delivered at Roots, a Black-owned birth center, on the experience of client autonomy and respect.

**Methods:**

To investigate if there was an association between experiences of autonomy and respect for Roots versus the national Giving Voice to Mothers (GVtM) participants, we applied Wilcoxon rank-sum tests for the overall sample and stratified by race.

**Results:**

Among BIPOC clients in the national GVtM sample and the Roots sample, MADM and MORi scores were statistically higher for clients receiving culturally-centered care at Roots (MADM p < 0.001, MORi p = 0.011). No statistical significance was found in scores between BIPOC and white clients at Roots Birth Center, however there was a tighter range among BIPOC individuals receiving care at Roots showing less variance in their experience of care.

**Conclusions for Practice:**

Our study confirms previous findings suggesting that giving birth at a community birth center is protective against experiences of discrimination when compared to care in the dominant, hospital-based system. Culturally-centered care might enhance the experience of perinatal care even further, by decreasing variance in BIPOC experience of autonomy and respect. Policies on maternal health care reimbursement should add focus on making community birth sustainable, especially for BIPOC provider-owners offering culturally-centered care.

## Introduction

Currently, more than 98% of birthing people in the United States deliver in hospitals (MacDorman & Declercq, 2019). Of grave concern are recent findings that Black, Indigenous and people of color (BIPOC) report mistreatment in the hospital two to three times more frequently than their white counterparts (Sakala et al., 2018; Vedam et al., 2019). One in ten respondents in the national Listening to Mothers III survey reported harsh language and rough handling when giving birth in the hospital, and Black individuals were more likely to report poor treatment (Declercq et al., 2014). Disrespectful treatment leads to numerous poor outcomes for birthing people and their infants and has repercussions on patient’s willingness to engage with the healthcare system beyond the pregnancy. Conversely, respectful, relationship-centered care has the potential to improve birth outcomes including fewer interventions, improved communication, and empowerment of the birthing person (Altman et al., 2019; Bohren et al., 2019; Larson et al., 2020; Turpel-LaFond, 2020). In the Listening to Mothers—California study (Sakala et al., 2018), the authors found that Black birthing individuals wanted options for community birth centers and midwifery care but had the least access to it. In their recent systematic review of the evidence on effects of race and racial concordance on care, Shen et al. (2018) found that Black patients consistently experienced poorer communication quality, information-giving, patient participation, and participatory decision-making than white patients.

The Giving Voice to Mothers study (here forward referred to as GVtM) (Vedam et al., 2019) examined experiences of childbearing care among two national cohorts of individuals in the United States: those choosing to give birth in community birth settings (home or free-standing birth center), and those birthing in hospitals. This study found that 28.1% of women in hospital settings versus 5.1% of women in community settings experienced some type of mistreatment (Vedam et al., 2019). Researchers report that Black individuals have the lowest scores for autonomy in decision-making and the least access to desired care including racially concordant care and midwifery care (Attanasio et al., 2017). In the national Strong Start initiative led by the Centers for Medicare and Medicaid Services, the Health Resources and Services Administration, and the Administration on Children and Families, researchers found that birth center care is associated with reduced racial and ethnic disparities in perinatal outcomes (Urban Institute, 2018). For example, reduction in preterm birth, and rates for low-birthweight infants for Black individuals receiving care at birth centers was 6% as compared to the national rates of 13.7% (Alliman et al., 2019).

Efforts to achieve birth equity have often overlooked community-based settings, such as birth centers. Black birthing people, facing the highest morbidity, mortality, and rates of infant loss, voice strong desires for birth care at home and in birth centers with culturally congruent providers (Attanasio et al., 2017; Sakala et al., 2018). Black midwife-owned community birth centers, offering culturally-centered care may be a solution to these identified problems (Alliman et al., 2019; Hardeman et al., 2020a, b; Karbeah et al., 2019). Midwifery care delivered in community birth centers has been found to improve patient experience and birth equity for Black birthing people (Sakala et al., 2018; Vedam et al., 2019).

Currently, more than 345 freestanding birth centers in the United States exist, which is a growth of 76% since 2010, yet only eleven (3%) are owned and operated by BIPOC individuals (Research and Data, 2020). Presently, the U.S. midwifery workforce is over 90% white (Fullerton et al., 2015; Research and Data, 2020). Because so few community birth centers are run by midwives of color, very little is known about how culturally-centered care might impact individuals’ peripartum experiences of autonomy and respect in their care, much less how it might improve clinical outcomes. To address this gap, we explore how a community birth center, focused on the needs of the Black community, and offering culturally-centered care to all its clients (*client* is preferred terminology over *patient* as it implies the individual’s choice in care and a state of wellness rather than illness), impacts the experience of respect and autonomy of both BIPOC and white clients.

## Methods

The aim of this study was to summarize and compare experiences of maternal respect and autonomy during childbearing care between two similar samples: birthing people receiving care with Roots Community Birth Center (here forward referred to as Roots) and the national sample of those in community birth settings in the GVtM study. Roots is owned by a Black midwife and is a culturally-centered community birth center in Minneapolis, MN. Binary variables of BIPOC and white were used in this analysis, BIPOC meaning Black, Indigenous, and any person who does not identify as white and/or individuals who identify as both white and another race, and white including only those participants that self-identified solely as white.

### Study Setting

Roots was created to meet the needs of Black birthing people in the Camden neighborhood of North Minneapolis where it is located. Camden is 60% BIPOC-identified, with most residents identifying as Black. Thirty-eight percent of households in Camden make less than $35,000 in annual income. The culturally-centered model of care at Roots includes 13–15 prenatal visits, with no visits scheduled for less than 30 min (Hardeman et al., 2020a, b). Clients are required to attend four group prenatal classes during prenatal care. When complications occur, warm hand-offs between Roots and hospital midwives are the standard of care and, when possible, care postpartum is transferred via warm hand-off back to the Roots midwives. Postpartum care includes lactation support with three home visits in the first week, and clinic visits at week two, four, and six. Culturally-centered care at Roots means acknowledging the client’s cultural community as a strength, providing racially concordant care as able, aligning with a mission of racial justice, and providing physically and emotionally safe care (Almanza et al., 2019). Clients at Roots, including Black birthing people, are considered experts in their own health and work alongside a midwife who provides medical expertise and person-centered decision-making (Hardeman et al., 2020a, b; Vedam, 2018).

### Study Procedures

Data for the analysis presented in this paper was collected as part of the *Birth Equity Project* (2016–2018) that examined the impact of culturally-centered care on the experiences of individuals receiving care at Roots and the motivations of midwives, doulas, and birth workers associated with Roots (Almanza et al., 2019; Karbeah et al., 2019). Employing a public health critical race praxis (PHCRP) approach (Ford & Airhihenbuwa, 2010), the research team utilized mixed methods to understand patient experience and provider motivation in peripartum care targeted to serve Black individuals in North Minneapolis.

Roots employed purposive sampling to distribute a postpartum survey to all individuals who had received care and/ or delivered their infants at Roots from its inception in the fall of 2015 through December 2018 (N = 360). Of these, 107 surveys were submitted (response rate of 29.7%) and 80 surveys were completed fully enough to use in this analysis. Participants who transferred to the hospital for care were also included in this study. Patients received the survey either in person before or after an appointment or via an email link. Study purpose and procedures were reviewed with potential participants and informed consent was obtained prior to participation either on paper or via the survey delivered via email. Clients did not receive an incentive for participation. Approval was granted by the University of Minnesota Institutional Review Board.

### Roots Survey Construction

The Roots postpartum survey developed as part of the *Birth Equity Project,* incorporated questions from two validated, national surveys: 1) the *Listening to Mothers* (LTM) survey—a national representative survey of mothers’ experiences and 2) the Changing Childbirth in British Columbia (Canada) study that used a participatory process to develop two scales designed to measure experiences of autonomy and respect during childbearing care (Vedam et al., 2018). The final survey included ten items from the LTM-III survey and 19 items from the Mothers Autonomy in Decision Making (MADM) scale and Mothers on Respect (MOR) index. Face validity of the Roots postpartum survey was established by reviewing survey questions with Roots owner and staff. The survey was piloted with a small sample (n = 15) of individuals who had recently given birth and clarifications and edits were made to the survey based on the pilot. Content validation was led by a study team member with expertise in the Listening to Mothers survey items along with Roots owner regarding their model of care. The Roots postpartum survey was open between June 2017 and December 2018.

The Giving Voice to Mothers (GVtM) survey was also a purposive sample and was administered to individuals who experienced pregnancy in the U.S. between 2010 and 2016 (Vedam et al., 2019) and included the MADM scale and MOR Index. The GVtM online survey with embedded consent was open from March 2016 to March 2017 (Vedam et al., 2019) and during this time 2700 people from all 50 states participated in the GVtM survey, including 244 people who planned to give birth in a free-standing birth center (Vedam et al., 2019). This sub-sample of 244 was included as the comparison group in the current analysis. Detailed information on survey construction, recruitment, and sample characteristics for the GVtM survey national study are published elsewhere (Vedam et al., 2019). The Behavioral Research Ethics Board at University of British Columbia approved the study.

### Measures

The Mother’s Autonomy in Decision Making (MADM) scale and Mothers on Respect index (MOR) are person-centered, validated, and reliable tools to capture the experiences and perceptions of birthing people in this comparative analysis (Vedam et al., 2017a, 2017b, 2018; Feijen-de Jong et al., 2020; Rubashkin et al., 2017; Vedam et al., 2017a, 2017b).

#### MADM

The MADM scale measures an individual’s autonomy in decision-making while receiving maternity care. This scale includes seven items that describe the nature of the respondent’s involvement in the decision-making process during their course of care, including whether they were apprised of risks and benefits, if they had time to consider options, and whether their choices were respected. Scores range from 7 to 42, with each of the 7 items having 6 Likert-response options. Low scores indicate loss/lack of autonomy in decision-making.

This scale has been evaluated as having strong psychometric properties, has been validated for variety of provider types and service users, and is appropriate to use with clients receiving care at a community birth center (Vedam et al., 2017a, 2017b, 2018; Feijen-de Jong et al., 2019; Rubashkin et al., 2017).

#### MOR

The Mothers on Respect (MOR) index measures experiences of respect while receiving maternity care. It has been validated in both a 7 item and 14 item version, but this study used the 14-item version. The MOR index includes three items about how comfortable the respondent felt when asking questions, declining care and accepting their provider’s recommendations. One item asks about perceived coercion during care. One item addresses choice in care options. Two items measure the degree to which individuals felt their personal and cultural preferences were respected during their course of care. Three items ask about whether participants held back questions because their care provider seemed rushed, they wanted care that was different from what their midwife or doctor recommended or because they thought their doctor or midwife might think they are being difficult. Four items ask about whether childbearing people felt that they were treated poorly by their care providers because of their race, ethnicity or cultural background, sexual orientation or gender identity, type of health insurance or because of a difference in opinion with their care provider. MOR items that measure negative experiences were reverse scored so that high scores on the MOR consistently denote respectful experiences. Scores range from 14–84 as each item is measured on a 6-point Likert scale. Low scores indicate less respectful care, including less culturally congruent care. Psychometric analysis demonstrates that the MOR is a reliable patient-designed indicator of the nature of interactions between service users and providers of pregnancy and childbearing care (Vedam et al., 2017a, 2017b).

In the Roots postpartum survey one MADM item (*I was able to choose what I considered to be the best care options)* and one MOR item (*I felt pushed into accepting the options my doctor or midwife suggested)* were deemed redundant with other items from the survey and hence were not included. For consistency, these items were also omitted when creating MADM and MOR scores for the comparison group. Therefore, in our study, MOR included 13 items and MADM included six items. MOR and MADM scores were calculated for participants who had all items. If an item was skipped, the overall score was considered missing. Because the original scales were altered for the purpose of this study, internal consistency reliability had to be established for the 6 item versions of MADM and 13 item version of MOR. The Cronbach’s alpha for the MADM items was 0.98 and for the MOR items it was 0.95 for Roots participants; GVtM participants had an alpha of 0.93 for the MADM items and 0.94 for the MOR items.

### Analysis

We compared MADM and MOR scores between white and BIPOC individuals with the goal of understanding how culturally-centered care delivered by a community birth center might influence the experience of autonomy and respect. We summarized participant characteristics for Roots and the GVtM birth centers using descriptive statistics. To investigate if there was an association between experiences of autonomy and respect for Roots versus GVtM participants, Wilcoxon rank-sum tests were performed. The same analyses were performed to investigate if there was an association between race and outcomes, and between site and outcomes for people of color. Overall MADM and MOR scores are presented using boxplots for Roots and GVtM birth centers, BIPOC and white participants at Roots, and BIPOC participants at Roots and GVtM birth centers. All reported p-values are two-sided and a significance level of 0.05 was used. Statistical analyses were performed using R version 3.6.2 and SAS (version 9.4, SAS Institute Inc., Cary, North Carolina).

## Results

### Participant Characteristics

Participant demographics of Roots and GVtM participants are found in Table 1. Of the 80 Root clients who were consented to participate in this study, over a third identified as people of color (n = 26, 34.2%). The GVtM sample (n = 244) also consisted of a third of respondents identifying as people of color (n = 80, 33.3%), which was not significantly different from the Roots sample (p = 0.888).

**Fig. 1:**
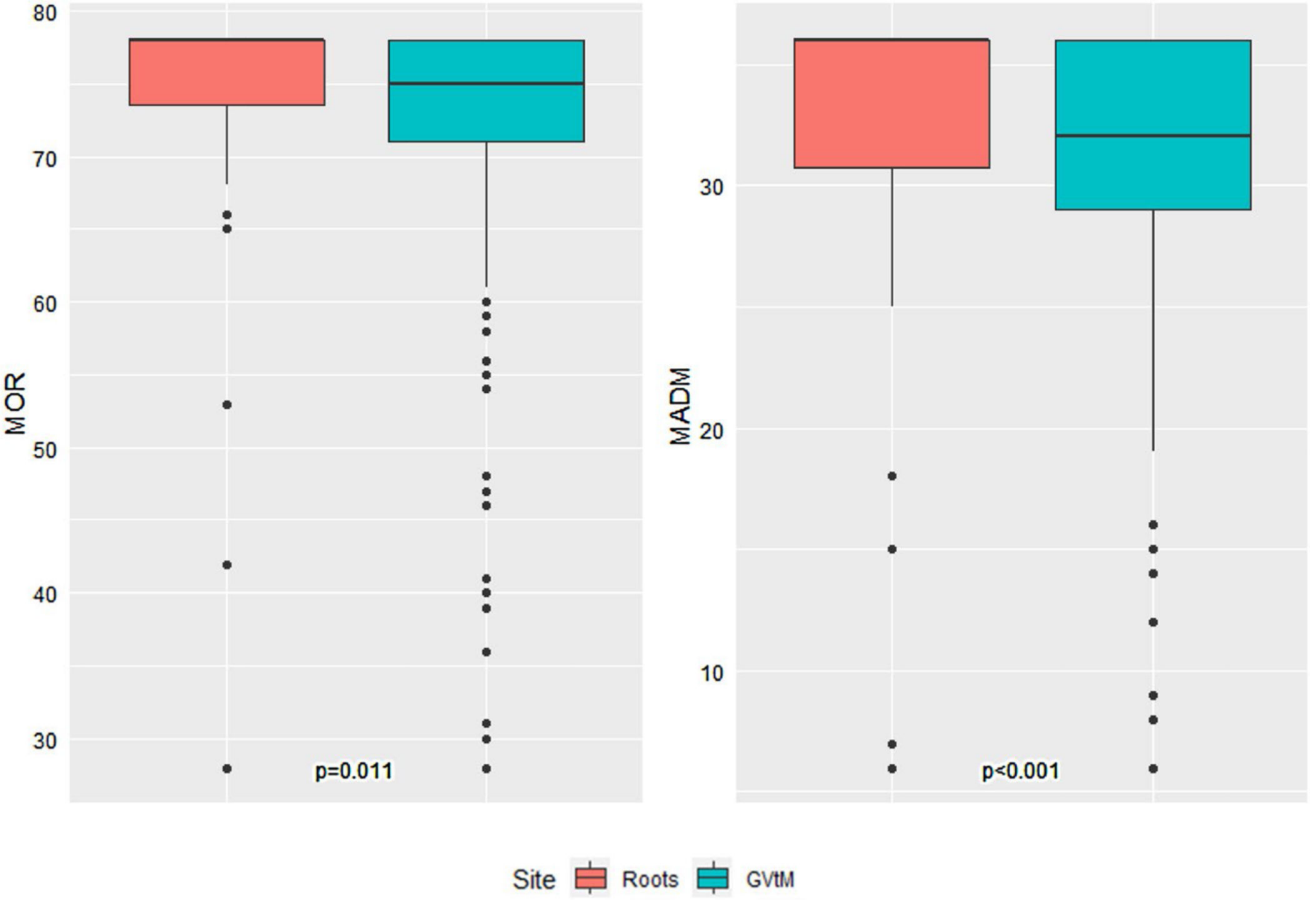
Comparison of MADM and MOR scores between Roots and GVtM birth centers (N = 324). Roots sample had 13 participants with missing MOR scores and 8 participants with missing MADM scores, while GVtM sample had 41 participants with missing MOR scores and 22 participants with missing MADM scores

The association between Roots and GVtM MADM and MOR scores is summarized in Figure 1. Roots participants reported significantly higher total MADM and MOR scores. In Roots the median score was 36.0 and among people receiving care at GVtM, the median MADM score was 32.0 (p < 0.001). Similarly, median MOR scores for Roots was 78.0 and GVtM was 75.0 (p = 0.011).

**Fig. 2:**
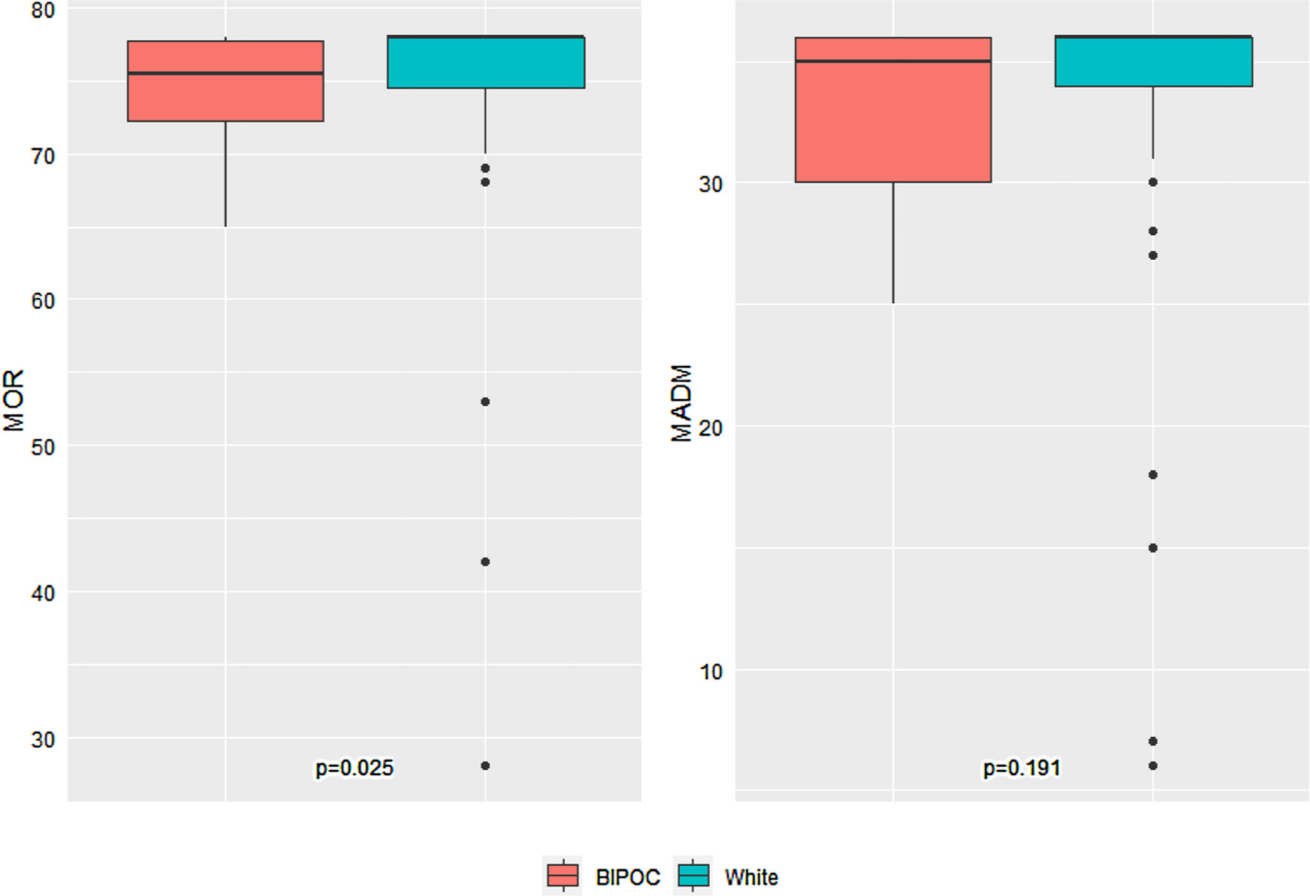
Comparison of MADM and MOR scores between BIPOC and white participants at Roots (N = 76). The Roots BIPOC sample had 4 participants with missing MOR scores and 2 participants with missing MADM scores, while the Roots white sample had 7 participants with missing MOR scores and 4 participants with missing MADM scores

The association between race and MADM and MOR scores at Roots birth center is summarized in Figure 2. No statistical difference was found in MADM scores between BIPOC and white individuals receiving care in the total combined Roots and GVtM sample. However, there was a significant difference in MOR scores between BIPOC and white individuals (median score 75.5 vs 78.0, p = 0.025). Ranges were noted to be consistently larger for white clients (MADM = 6.0–36.0, MOR = 28.0–78.0) than for BIPOC clients (MADM = 25.0–36.0, MOR = 65.0–78.0) which indicates more variation in experience.

**Fig. 3:**
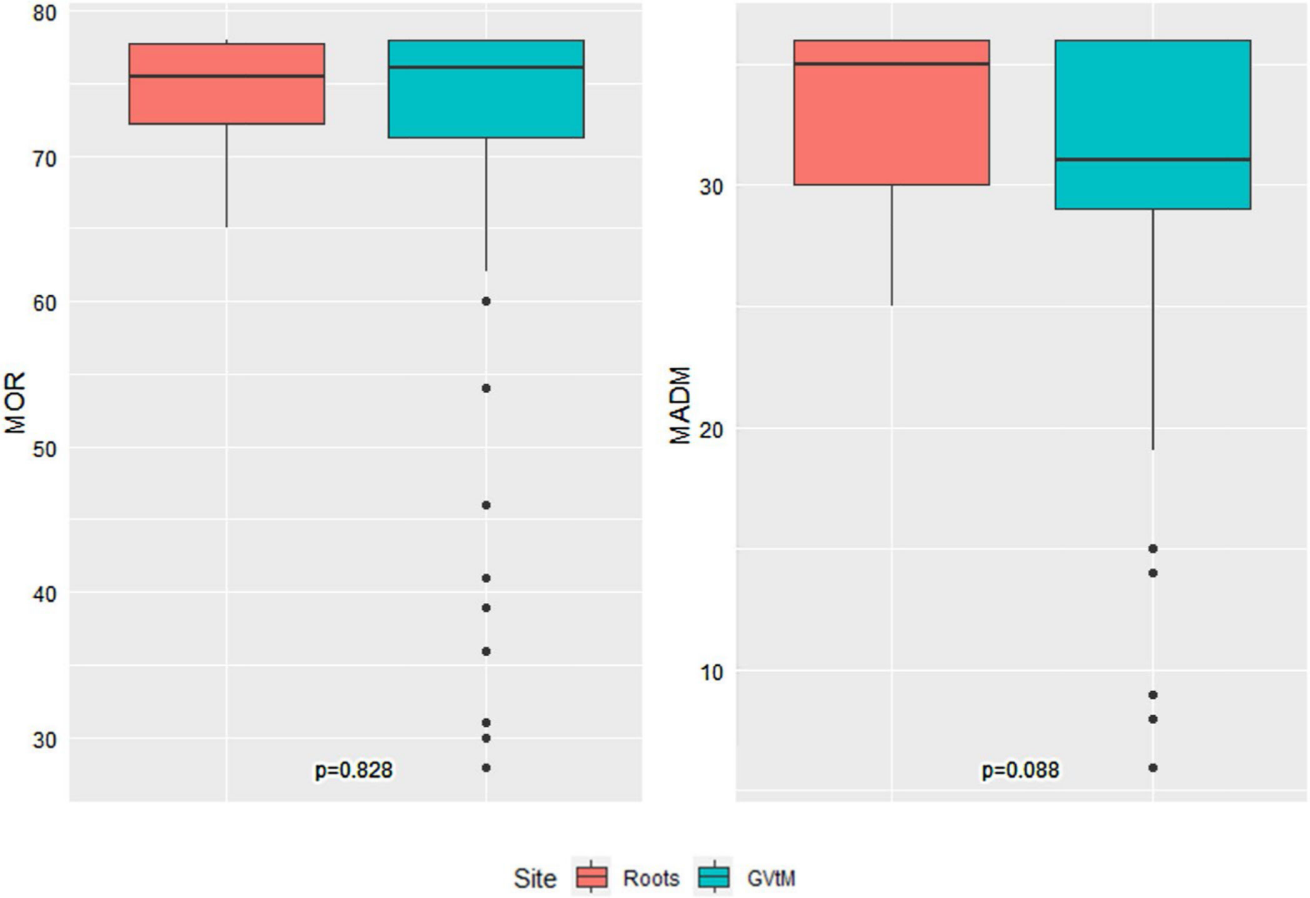
Comparison of MADM and MOR scores between BIPOC participants at Roots and GVtM birth centers (N = 106). The Roots BIPOC sample had 4 participants with missing MOR scores and 2 participants with missing MADM scores, while the GVtM BIPOC sample had 14 participants with missing MOR scores and 3 participants with missing MADM scores.

The association between BIPOC participants for each birth center and MADM and MOR scores is summarized in Figure 3. No statistical difference was found between BIPOC individuals receiving care at Roots versus those receiving care at GVtM birth centers. However, once again the range was tighter among BIPOC individuals receiving care at Roots (MADM = 25.0–36.0, MOR = 65.0–78.0) versus those from the GVtM study (MADM = 6.0–36.0, MOR = 28.0–78.0).

## Discussion

To make best practice guidelines, recent studies (Hardeman et al., 2020a, b; Niles et al., 2020; Shen et al., 2018; Zampas et al., 2020) have attempted to identify what elements of maternity care improve the experience and outcomes of BIPOC birthing individuals. Our unique analysis took previous findings on the benefits of birth center care, including more equitable outcomes (Alliman et al., 2019; Stoll et al., 2019; Urban Institute, 2018), a step further to identify how receiving culturally-centered care at a Black-owned birth center links to client experience. Birth center utilization is on the rise for all racial and ethnic groups, yet the largest increase in utilization has been among non-Hispanic white women who already comprise the greatest number of birth center clients and providers (MacDorman & Declercq, 2019; Stapleton et al., 2013). We found that care provision at Roots a culturally-centered model of care, is associated with statistically higher levels of autonomy and respect for all clients, regardless of race. Respect and autonomy in decision making are two elements in the World Health Organization’s quality-of-care framework for maternal and newborn health (Zampas et al., 2020).

There were high levels of respect and autonomy and less variance in experience for BIPOC individuals at Roots as compared to participants in the GVtM study, suggesting that taken alone the birth center model is still missing elements necessary to create the experience of autonomy and respect for BIPOC individuals. White individuals at Roots experienced a larger variance in experience; some white clients at Roots scored on the lower end of the MADM and MOR scales whereas none of the BIPOC clients did. This finding may suggest an element of inherent trust or comfort in the care provided by one’s own affinity group, however, for BIPOC individuals offering this type of care is difficult in a system with so few BIPOC midwives (Fullerton et al., 2015). These results are similar to findings from Shen et al. (2018) who found that racial concordance was a predictor of better client-provider communication, indicating certain elements cannot be learned. Thus, care across birth settings for BIPOC individuals may differ by the model of care and identities of providers at each setting. These findings are unsurprising in a country publicly grappling with a history of racism still institutionalized across public sectors, including healthcare, today (Hardeman et al., 2020a, b).

While researchers conduct ongoing work to understand how health care might better engage and serve those in the BIPOC community (LaVeist & Nuru-Jeter, 2002; Stevens et al., 2003), until recently, there has not been empirical evidence linking improved health outcomes to racially-concordant care. The first investigation of this connection found that when Black newborns are cared for by Black physicians, their likelihood of death is cut in half. (Green-wood et al., 2020). This groundbreaking study is compelling in that communication between patient and provider does not exist (as one participant is non-verbal), suggesting that receiving racially-concordant care has a protective effect on birth equity in and of itself. It is difficult to know if positive effects of racial concordance are due to trust found inherently in affinity groups, or if they are due to value being given to a client’s cultural norms and identity, as is the case in culturally-centered care.

Previous findings suggest that midwifery care delivered in birth centers is protective against experiences of discrimination (Alliman et al., 2019; Vedam et al., 2019). A recent qualitative study of 20 midwives (of which over a third were BIPOC) practicing in two publicly funded hospitals in New York City described the midwifery model as caring for the social body, and with care rooted in one’s economic, political, physical, and historical position (Niles et al., 2020). Further, recent research demonstrates that even in publicly funded hospital systems, if able to practice to their full scope and supported by institutional power structures, midwives can provide culturally-centered, relationship-based, personalized care to BIPOC individuals that results in optimal outcomes. (Niles et al., 2020). While our analyses suggest that culturally-centered care enhances BIPOC peripartum experience, racially-concordant care is something that can be actualized only when there are more BIPOC midwives and BIPOC-run birth centers to study. Community birth centers staffed by BIPOC providers warrant renewed attention, investment, and funding to improve health equity (Alliman et al., 2019; Hardeman et al., 2020a, b).

### Limitations

This study evaluated the impact of culturally-centered care using a quantitative survey instrument, and comparative data from a national survey. Capturing some qualitative data, in addition to the survey data, would have offered further insight on the characteristics of care that affect how respect and autonomy are experienced in these spaces. A second limitation of this study is the small size of our analytic sample from Roots. This limits the generalizability of findings. Culturally-matched care as an intervention to improve client experiences and perinatal outcomes should be tested using longitudinal study designs that control for baseline differences of childbearing people who receive culturally-matched care versus those that do not. Exposure to culturally-matched care in the control group and other factors that might influence group differences in study outcomes should be carefully measured and controlled for. In future studies, sample size calculations ought to determine the minimum number of participants needed to detect significant differences in the primary outcome. Notably, only a third of both the Roots and the GVtM samples identified as BIPOC. Hence, from theoretical and analytic perspectives, our small sample sizes, and our decision to combine all BIPOC respondents may obscure unique racial or cultural experiences. Additionally, there is a lack of consensus on how culturally-centered care is defined. Further studies evaluating data from focus groups with childbearing people who have experienced culturally-centered care would advance our understanding of the elements of culturally centered care.

Our work was conducted in collaboration with the sole Black-owned and operated community birth center in one region. Future studies might seek to administer the MADM and MOR scales in the other 10 BIPOC-owned birth centers around the country to validate similar findings for BIPOC individuals receiving culturally-centered care. Funding should be directed to larger scale follow-up studies that can control for baseline differences of participants. Emerging research on the impact of models of care on quality of care should disaggregate analyses by race and ethnicity of both clients and providers, and their impact on client experience of respectful care. One avenue that could be considered is more widespread and consistent implementation of client feedback on their experiences of respect and autonomy. This would help us to understand if increased client experience is related to culturally-centered care, or rather, related to respectful maternity care. Respectful care is teachable through tools that incorporate shared decision making, upholding patient autonomy, and respectful language. Above all, more funding is needed to grow the pool of midwives in practice offering culturally-centered care and creative solutions to improving the experience of care among BIPOC families.

Lastly, it is important to note that community birth centers across the country are staffed and owned by Certified Midwives (CMs), Certified Practicing Midwives (CPMs) and Certified Nurse Midwives (CNMs), all of which are a part of filling the care gap.

## Conclusion

Our findings show that both BIPOC and white clients report very high autonomy and respect scores at Roots, significantly higher than a larger comparison sample. Results from this small exploratory study suggest that culturally-centered care at a birth center, confers benefit and may improve birth equity and the experience of BIPOC families during the transformative and critical time of childbearing.

## Figures and Tables

**Table 1:** Summary of demographics for Roots and GVtM participants (N = 324)

Variable	Roots participants (N = 80)	GVtM participants (N = 244)

*Age*, n (%)		
Number missing	8	18
18–24	8 (11.1%)	16 (7.1%)
25–29	17 (23.6%)	59 (26.1%)
30–34	28 (38.9%)	97 (42.9%)
35 and older	19 (26.4%)	54 (23.9%)
*Race*, n (%)		
Number missing	4	4
Black or African American	10 (13.2%)	38 (15.8%)
White	50 (65.8%)	160 (66.7%)
Latina	-	28 (11.7%)
Asian	4 (5.3%)	7 (2.9%)
Indigenous	-	6 (2.5%)
Other person of color	1 (1.3%)	1 (0.4%)
More than 1 race	10 (13.2%)	-
African	1 (1.3%)	-
*Ethnicity*, n (%)		-
Number missing	5	
Hispanic or Latina	6 (8.0%)	
Not Hispanic or Latina	69 (92.0%)	
*Household income*, n (%)		
Number missing	5	49
Under $20,000	-	12 (6.2%)
$20,000-$49,999	-	56 (28.7%)
$50,000-$99,999	-	70 (35.9%)
Over $100,000	-	57 (29.2%)
$15,000 or less	3 (4.0%)	-
$15,001-$37,000	16 (21.3%)	-
$37,001-$67,600	27 (36.0%)	-
$67,601-$102,000	20 (26.7%)	-
Over $102,000	9 (12.0%)	-
*Main source of payment for maternity care*, n (%)		
Public		42 (17.2%)
Private		131 (53.7%)
Out of pocket		42 (17.2%)
Other		29 (11.9%)
*State where gave birth*, n (%)	-	
Number missing		8
Connecticut		31 (13.1%)
New York		28 (11.9%)
Texas		30 (12.7%)
Other		147 (62.3%)
*Parity*, n (%)	-	
Nulliparous/primiparous		114 (46.7%)
Multiparous		130 (53.3%)

## Significance

National studies have found birth center care is associated with reduced racial and ethnic disparities, yet very few are owned by BIPOC midwives (3%). Even when receiving birth center care, white individuals consistently report higher scores on autonomy and respect, suggesting that barriers may prevent BIPOC individuals from receiving care that meets all their needs. Findings in this study suggest that there is less variability in the experiences of BIPOC individuals when they receive culturally-centered care. Further, statistically significant higher scores on autonomy and respect suggest that culturally-centered care might improve overall experiences of autonomy and respect regardless of race.
